# Parents’ Perceptions of Teachers’ Authority and Parental Involvement: The Impact of Communality

**DOI:** 10.3389/fpsyg.2022.908290

**Published:** 2022-06-16

**Authors:** Yael Fisher, Ravit Refael Fanyo

**Affiliations:** ^1^Hemdat Academic College, Netivot, Israel; ^2^Achva Academic College, Arugot, Israel

**Keywords:** teachers’ authority, parental involvement, communality, elementary schools, SEM

## Abstract

This study aimed to examine how the level of communality (communal affiliation) affects parents’ perception of children attending public elementary schools, the concept of teacher authority, and the concept of parental involvement. The study population consisted of 300 parents living in various parts of Israel who agreed to complete a self-reporting anonymous questionnaire. The questionnaire comprised three subsections, two of which were based on previous studies: Scale of parents’ perception of “parental involvement,” which included 44 items, Cronbach’s alpha value was 0.90.; The Scale of parents’ perceptions of the concept of “Teacher’s Authority,” which included 25 items, Cronbach’s alpha value was 0.79; and one was composed primarily for the current study, the Scale of parents’ perception of “Communality Level” which included 19 items, Cronbach’s alpha value was 0.88. The findings were analyzed using structural equation models (SEM). Applying these measures to the current study rendered the following results: RMSEA = 0.007, TLI = 0.995, CFI = 0.99, NFI = 0.904, df = 16, χ^2^ = 16.266, *p* = 0.435. Hence, the value of 1.01 ($\frac{{\text{x}}^{2}}{d\,\text{f}}$) < 3, the TLI and CFI > 0.95. The research findings indicated that a high level of communality (communal affiliation) among parents predicted high levels of perceived teachers’ authority (β = 0.27) and parental involvement (β = 0.30). By contrast, it was also found that living in the same residential characteristics as the teachers predicted low levels of both perceived teacher authority (β = −0.18) and parental involvement (β = −0.20). As regards the theoretical aspects, it adds a new layer to educational research about the variables that affect perceptions of teacher authority, an issue that has received little attention in the research literature. In terms of its practical applications, the model can help education systems in general and schools, in particular, to formulate policies and take steps to improve the ever-important relationship between the school and the parents. Furthermore, the model clarifies our understanding of and ways to strengthen the teacher’s authority.

## A Review of the Literature

### The Teacher’s Authority

“Authority” refers to the likelihood that one will be obeyed by others voluntarily. It indicates the right of such a person to give commands and teach; it also depends significantly on the legitimacy granted by others to the authority figure. In other words, authority is the connection between commands and obedience, which is based on the leader’s legitimacy and the voluntary obedience of the followers. This relationship between the two parties is grounded in a moral order, including common goals, values, beliefs, and norms (Chafi et al., 2016).

Authority is affected by ethnicity, social status, and political and cultural contexts (Pace, 2003). The attitude toward authority is a cultural characteristic and indicates how members of a particular culture emphasize equality vs. hierarchy. Members of a society with a low degree of obedience to authority are typically independent, free, and aspire to equality between the power holders and the others. In cultures with a high degree of obedience to authority, members are typically conformists and recognize the legitimacy of inequality and the implicit struggle between power holders and those without (Tubin and Leese, 2013).

In education, authority refers to the relationship between teachers and students as a hierarchical relationship between unequal parties who participate in the school-based educational endeavor, wherein one party (the authority figure) determines a range of actions or knowledge for the other party (the abider), who may follow through or refuse to act as indicated by the authority figure. This relationship runs the gamut between making demands on students and creating emotional ties with them to encourage learning. Teachers must convince students to cooperate, and students must agree and be prepared *to* receive what is taught. This relationship includes elements of freedom, power, and legitimacy, which students afford their teachers and which teachers view as inherent *to* their role. These elements can be interlaced in various ways, and their manifestations depend on the character of the school (Pace, 2003; Pace and Hemmings, 2007; Goodman, 2010).

Educational authority is based on teachers’ legitimacy and legal role. It is there a legitimate right to educate and teach. It is their responsibility and that of the school to educate students to benefit the schools and society in general.

Furthermore, teachers’ authority is based on their knowledge and expertise in the field they teach, their ability to dictate the pace of learning in the classroom, their evaluation of the students, and their ability to maintain discipline. Their authority is also related to their personal life experience, their experience in teaching, their expertise in additional fields, and their belonging to a prestigious, influential, and respected (Wenren, 2014).

Some consider the characteristics of teachers’ authority similar to those of parental authority. An authoritative teacher, just like an authoritative parent, combines a high degree of care for one’s students, which is expressed in a warm relationship, along with high academic demands and expectations from one’s students (Dever and Karabenick, 2011; Risanger Sjursø et al., 2019).

In recent decades, the teacher’s authority in the classroom has begun to deteriorate, especially in modern Western societies. In Israel, the deterioration in teachers’ authority was greatly affected by one of the noticeable characteristics of Israeli society, namely, a measured rejection of formal authority. As agents of socialization, schools constitute an arena into which these cultural values are implanted (Tubin and Leese, 2013; Noy, 2014; Omer and Maimon, 2019).

Moreover, teachers have lost their authority as knowledge sources. In an era characterized by high accessibility and multiple ways to obtain information, the teacher is no longer a unique source of power on knowledge and information (Raviv et al., 2003). Likewise, integrating electronic and communication technologies into computer-based education, whether in the classroom or through distance learning, further eroded familiar forms of teachers’ authority. Furthermore, Students’ technological literacy is much higher than their teachers. As a result, students tend to question the authority of their teachers.

We cannot ignore the fact that in the Western world in general and in Israel, parents’ increased involvement in the education system and their attitudes toward teachers contribute to the further erosion of teachers’ status and authority. In contrast to teachers’ total support in the past, legitimate criticism can quickly become inappropriate intervention and completely shatter Students’ perception of the teacher’s authority and status. Some parents do not consider teachers a pedagogical authority or someone to consult with. More often than not, they share these sentiments with their children (Israeli National Program of Education, 2005; Ben-Peretz, 2009; Gilat and Vangarovitz, 2018). Likewise, written and broadcast media often paint teachers negatively (Noy, 2014).

The perception of teachers’ authority is different in countries in the Far East, such as China, South Korea, and Hong Kong. There, the culture is authority-oriented with a clear hierarchical structure, and there is a well-defined hierarchy between teachers and students. Two golden rules guide students in these cultures: the obligation to respect the teacher and the teacher’s knowledge and the truth being taught. Undermining or criticizing a teacher’s ability is perceived as impolite and unacceptable behavior (Chan and Chan, 2005; Lee and Kim, 2017).

Nowadays, researchers agree that education without authority leads to negative results. It has been shown that children educated in a framework devoid of authority had a low threshold for frustration and a poor self-image; they dropped out of the educational framework and were exposed to numerous risks (Omer, 2018). The other side of this equation demonstrated that teachers’ authority positively affected students. As authority figures, the teachers had high expectations and demands of students while exhibiting high warmth and care toward them. In these cases, students demonstrated a higher degree of interest in their studies, attained better academic achievements, and demonstrated lower levels of violence and bullying (Dever and Karabenick, 2011; Risanger Sjursø et al., 2019). Hence, it is not surprising that there has been a growing discourse on rehabilitating teachers’ authority.

Some scholars claim that in the postmodern era, we cannot expect that teachers’ authority will be restored to them by their position; instead, its rehabilitation can be obtained via other sources and using different approaches in the classroom (Sharmer, 2005; Omer, 2018). One way to restore educational authority is to ensure that students view their teachers as role models to be imitated and seek to internalize the values they represent. To this end, teachers need to be appreciated by their students as skilled professionals who set reasonable and well-founded limits instead of as an outside force that seeks to impose its ways on them. In the dialogue that takes place between teachers and students as they attempt to clarify disagreements and address reservations, students will have access to their teachers’ thoughts and views and thus will be able to appreciate their attitudes and internalize the values and norms underlying the instructions and limits conveyed by their teachers. Likewise, acting as personal role models, practicing and demonstrating the values they wish to inculcate in their students is a path that will help restore and strengthen their authority in the classroom (Amit, 2005).

The professional literature suggests building trust as an alternative to authority. The culture of trust views teachers and students as shareholders instead of the approach according to which the students are the teachers’ subjects. In the context of trust, the two parties are mutually dependent and rely on each other to attain their respective goals. When a mutual agreement is viewed by both parties, they find it easier to establish positive interactions and relationships. Building trust between teachers and students can be attained by engaging in joint learning and developing a discursive culture. They will gain in-depth knowledge and understanding of each other (Sharmer, 2005).

Another approach considers the decline in teachers’ authority in the context of knowledge authority and addresses the reconstruction of authority from this respect. The teacher’s role should help the students attain knowledge on their terms and based on their unique abilities. This pedagogical approach emphasizes that education in this day and age is not limited to transferring knowledge or retaining knowledge. Instead, education no means offering students the opportunity to develop their skills and apply and organize knowledge using effective strategies. Thus, classroom studies should be based on dialogical teaching, guidance, and provision of resources and support for the learners to advance their ability to study independently and prepare them to meet the challenges of the current day and age. In this manner, the teacher’s role can be reformulated and redefined to correspond to today’s Western society (Levi-Feldman, 2020).

### Parental Involvement

In recent decades, the concept of “parental involvement” has been the subject of widespread attention from educators, educational researchers, policymakers, and parents and parent organizations in Israel and throughout the world. Despite the numerous definitions of this concept, there is a broad acceptance that it defines the reciprocal relationships between parents and the educational institution and parents’ investment in various resources related to their children’s education. Researchers have characterized parental involvement through multiple actions and activities, which can be active or passive, and manifest on two separate planes. One such plane is the organizational level, i.e., parents’ activities within the school, which are directly related to the school, such as formulating a school policy, communicating and meeting with teachers, and participating in school activities and workshops. A second plane refers to the level of the individual child, that is, joint activities that parents engage in along with their children within the school and which are related to learning processes, for example listening to children read out loud or supervising homework preparation (Friedman and Fisher, 2002; Hornby and Lafaele, 2011; Ice and Hoover-Dempsey, 2011; Wilder, 2014; Fisher, 2016).

In Israel, parental involvement developed and was perceived differently from one decade to the next. In the 1990s, there was an additional change in the concept, and the idea of parental involvement became prominent. This process resulted from changes in Israeli society and the transference of power from the central government to the parents and the community (Machter, 2001; Fisher and Friedman, 2009; Fisher, 2010).

Parents have different reasons for becoming involved in their children’s schools and study processes. Studies have shown that parents of children of all ages perceived school involvement as part of their role as parents and the responsibility for their children’s education. An additional reason is parents’ self-efficacy, as they consider themselves capable of helping and advancing their children’s studies. Also, children’s requests and wishes motivated parents to become involved in an attempt to address their children’s needs. A school that welcomes parental involvement also encourages this type of behavior. In Israel, it was found that parental involvement is also the outcome of the financial involvement of parents in funding educational programs. As a result, parents felt that they have the right to act as partners in the more crucial decisions (Barger et al., 2019).

Furthermo*re*, a strengthening of the democratic spirit in society over the *years* emphasized the freedom of individuals *to* select a particular worldview and the educational approach desired for their children. The student contributed *to* the perception that parents should be allowed *to* determine the type of education their children receive at school. *Moreover*, the erosion in teachers’ status and the lack of trust felt by the public and parents *toward* the education system led parents *to* become more involved in their children’s schools (Hoover-Dempsey et al., 2010; Friedman, 2011; Murray et al., 2014).

Other factors that may either encourage parents to intervene in their children’s schools or prevent parents from becoming involved include their awareness of and identification with the school. Identifying with the school’s importance and agreeing with its goals and mission, viewing the school as a place to acquire education and knowledge an equal opportunity is a pleasant place, engaging, and challenging, devoid of disciplinary problems. A safe place for the learners –these are all factors that affect the degree of parents’ involvement in the schools. Parents who identify strongly with these aspects will likely approve of the school’s values and norms and hence opt to become involved and active. By contrast, parents with a low degree of identification with the school are apt to reject the values and norms conveyed by the school to their children and take an oppositional stance. Another factor is parents’ awareness of the organizational culture of the school. Parental sensitivity to the dynamics at the school affect the degree of parental involvement in the children’s schools; parents’ awareness and concern about topics such as curricular contents and teaching approaches, the school’s function as an organization, the relationships between teachers and students, and among the students themselves, as well as issues of violence and discipline, create a desire to be informed and, hence, to get involved. A high degree of awareness means expressing interest and paying attention to what goes on at school; the absence of awareness means apathy and ignorance (Friedman and Fisher, 2002; Fisher and Friedman, 2009).

Another factor that influences parental involvement in schools is related to the family’s place of residence. It was found that parents whose children attended rural schools were more involved in their children’s schools than were parents of children who attended city schools. Moreover, parental involvement in rural areas may be perceived as more natural. The structure of rural communities, their size, parents’ affiliation and relationships with other residents within the community, and their social networks may strengthen the parents’ attachment to the school and their involvement. For the most part, the teachers and school principals in rural communities are community members, and people know them from this residential context. As a result, the school staff members typically share the same values and norms as those upheld by their Students’ families; they know their families and understand children’s apprehensions and difficulties. A better understanding helps for better relationships between the parents and the school and motivates parents to become involved. However, prior acquaintance with Students’ families can also lead to prejudices about particular students and become obstacles to parental involvement (Caplan, 1995; Bauch, 2001; Hornby and Witte, 2010; Lasater, 2019).

Notwithstanding the multiple reasons parents have for becoming involved in their children’s schools, many factors cause parents to avoid becoming involved in the schools or their children’s studies. Such impediments include parents’ income and socioeconomic status, life circumstances, and parental availability dictated by job requirements, the number of children, or other familial obligations (Murray et al., 2014; Barg, 2019). Additional obstacles identified through research were negative past experiences with an educational institution and parents’ poor sense of self-efficacy regarding their capacity to be helpful. Parents’ country of origin, language, culture, and ethnicity has been identified as obstacles to parental involvement in school. Furthermore, factors related to the student, the individual child, can prevent parental involvement. The child’s age, disciplinary problems at school, disagreements between parents and teachers about addressing the child’s needs, whether related to learning disabilities or exceptional talents, can impair the relationship between the parties (Hornby and Lafaele, 2011).

### Community and Communality

“Community” as a concept has numerous definitions and conveys various nuanced meanings. The topic has been studied in the context of anthropology, sociology, and psychology. Despite the multiplicity of definitions, scholars referred to the concept as relating to a group of people whose members have common interests and are involved in social and collective circles (Etzioni, 2000; Lehavi, 2006; Sadan, 2009). The concept of community differentiates between non-territorial and territorial communities. The former describes geographically dispersed groups whose members live among people who do not belong to the same community, for example, a business community, an ethnic community, an academic community, and an online community. The territorial community refers to a community with geographic boundaries, such as a neighborhood in a city or a small town where the population leads a communal lifestyle, conducting joint activities and sharing traditions (Theodori, 2005; Sadan, 2009).

In the current research literature, a “community” is described as a social system that maintains communication and reciprocity in most areas of life. It can be found in any form of settlement. A community is characterized by its social capital, which indicates a complex system of emotional relationships among individuals, defined based on one-to-one interactions and dynamics and complicated connections involving several people.

Another characteristic of a community is its territorial dimension, which enables face-to-face interactions and affects the quality of the relationships, the level of trust, the interchange, and the type of relationships among its members. Community members have an internal collective awareness that distinguishes them from their surroundings. They share values, norms, history, identity, and a commitment to a particular culture while addressing the shared day-to-day concerns. Notwithstanding, the new communities are pluralistic and advocate individualism and freedom combined with mutual responsibility and group commitment (Etzioni, 2000; Theodori, 2005; Lehavi, 2010; Shadmi-Wortman, 2017).

Lehavi (2010) added to the concept of community the term “gating,” which refers to the existence of at least one of two components: a formal system for determining who will be allowed to become members of the group and occasionally to decide who may be permitted to leave the group, and a physical boundary that prevents unwanted entities from entering the communal territory and its organizations.

The level of communality is a characteristic that indicates the essence of the community and the psychological aspect of living in this framework. Researchers have claimed that communality is measured in terms of human capital and is related to having a shared sense of identity unique interpersonal relationships among the community members and is affected by the level of trust and reciprocal relations among community members. In other words, it is measured in terms of the willingness of the individuals to act for the benefit of others, their desire to establish close relationships with other members of the community and involve them in their lives (Shadmi-Wortman, 2017). McMillan and Chavis (1986) identified several components of communality: socialization—the degree to which members of the community have a sense of belonging, understand the boundaries of the community, trust in and are willing to invest personally in the community; effect—the degree of cohesiveness in the community, which manifests in the effect that the individual has on the community and the impact the group has on the individuals and their actions; need fulfillment—the degree to which the community helps its members address personal and group needs; a shared emotional connection (Talò et al., 2014).

Studies have found that a high level of communality provides people living in the community with social and psychological resources that positively affect the individual living in it. The community contributes to an increase in the quality of life, improves the personal wellbeing and life satisfaction, helps cope with the stress of life, and even affects the quality of parenting of people in the community (Simons et al., 1997; Talò et al., 2014).

Israel’s territorial communities vary in size and functions, and a different collection of characteristics and contents constitutes its communality in any society. We find the traditional kibbutz on one end of the spectrum, which formed an entire community where the group carried out all functions within the exact geographic boundaries. Since 2001, the traditional definition of the kibbutz has changed; it is now referred to as the renewed kibbutz, i.e., in this framework, the property is shared by the group, work is attained on an individual basis, and production, consumerism, and education are shared equally.

In the kibbutz community, solidarity among members is a defining characteristic. At one end of the spectrum of communal life in Israel, one finds the communities where all the necessary life functions occur outside of the community’s geographic perimeter. The community serves as the geographic home, where members can be found at night and on weekends, so that essentially, the community serves as a framework for joint cultural events. Thus, addressing the number of functions within the community is the primary way to influence the degree and quality of communality (Lehavi, 2006; Shadmi-Wortman, 2017; Israeli Central Bureau of Statistics, 2020).

Another communal framework is called the “Moshav,” where production and marketing are other functions shared by its members and take place within the geographic confines of the community; however, consumption is managed on an individual basis in these communities. Yet another communal framework in Israel is a community settlement, and the settlement is a new type of rural/suburban population. The collaborative aspect is unrelated to work or finances but only living within a close-knit community (“communal settlements”). Some communal settlements function as a cooperative with no rights to agricultural lands. Yet, the members cooperate and are responsible for such functions related to production, consumption, municipality, and society (Lehavi, 2010; Israeli Central Bureau of Statistics, 2020).

In contrast to these communal frameworks, urban populations are characterized by individuality and personal freedom, the absence of kinship or any other emotional or close-knit relationship among the population members, and economic reciprocity. In recent decades, however, some neighborhoods have established themselves as communities to improve their quality of life, address safety issues, protect property value, and improve services to the neighborhood (Lehavi, 2006).

### Study Goals, Questions, and Hypotheses

The study aimed to the effect of the level of communality on the way parents of children enrolled in government-funded elementary schools perceive the concepts of teacher authority and parental involvement. From this goal, the following questions were derived:

(1) Will a relationship be found between the level of communality (communal affiliation) expressed by parents’ residential characteristics (living in different community frameworks) and their perceptions of parental involvement?
(2) Will a relationship be found between the level of communality (communal affiliation) expressed by parents’ residential characteristics (living in different community frameworks) and their perceptions of teacher authority?

**FIGURE 1:**
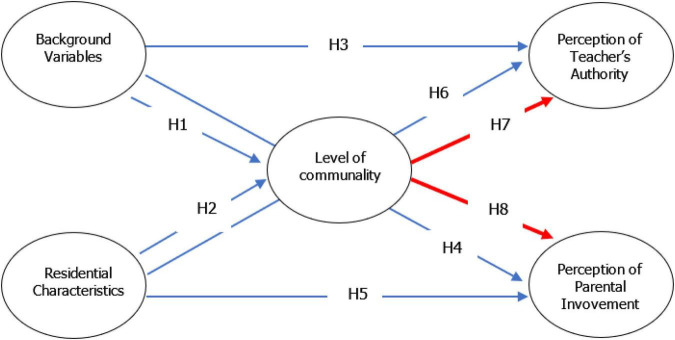
Conceptual framework.

The research literature does not indicate that the issue of the relationships between level of communality (communal affiliation) and perceptions of teacher authority and parental involvement has been investigated; hence, many hypotheses presented in the research model (see Figure 1) are exploratory.

### The Research Hypotheses

(1) The parents’ level of communality will predict their perception of both teachers’ authority and parental involvement (see H7 and H8 in Figure 1). This hypothesis is exploratory because the issue has yet to be studied.
(2) Parents’ background variables will predict their perceived level of communality (see H1). This hypothesis is exploratory because the issue has never been studied.
(3) The residential characteristics will predict parents’ perceived level of communality (see H1). This hypothesis is exploratory because the issue has never been studied.
(4) Parents’ background variables will predict their perceptions of teacher’s authority (see H3). In a study by Adetto (2012) a relationship was found between a parent’s age and the parent’s perception of teacher authority [*F*_(2,__84)_ = 5.767; *p* < 0.01]. Younger parents (*M* = 4.13) perceived teachers’ authority to be stronger than did either middle-aged parents (*M* = 3.66), [*t*(85) = −2.978: *P* < 0.01] or parents over age 55 (*M* = 3.47), [*t*(85) = −3.834; *P* < 0.01].
(5) Parents’ background variables will predict their perceptions of parental involvement (see H4). In the study by Fisher and Friedman (2009), a relationship was found between the level of parents at the involvement and their gender [*F*_(1,__1_,_228)_ = 7.01; *p* < 0.01], whereby women (*M* = 2.72) were more involved than men (*M* = 2.63). Another finding from the study was a relationship between parents’ level of active involvement and their level of education [*F*_(2,__1_,_266)_ = 6.88; *p* < 0.01], whereby parents with a higher level of education were found to be more active (*M* = 1.24) than were either parents with a low level (*M* = 0.94) [*t*(1,264) = 2.81; *p* < 0.B01] or those with an intermediate level (*M* = 0.98) [*t*(1,264) = −3.01; *p* < 0.01] level of education.
(6) The residential characteristics will predict parents’ perception of teachers’ authority (see H5). This hypothesis is exploratory because the issue has never been studied.
(7) The residential characteristics will predict parents’ perception of parental involvement (see H6). Although this was not directly studied regarding parental involvement, in Friedman and Fisher (2002) study, a relationship was found between parents’ residential framework and the extent to which they were able to identify with the school’s pedagogies, such that urban parents *M* = 2.91 agreed with the school’s pedagogical approach more strongly than did rural parents (*M* = 2.68) [*F*_(1,__197)_ = 2.82; *p* < 0.05]. As noted in the current study, agreeing with the school’s academic and pedagogical goals and mission is an essential element in parents’ perception of parental involvement.

## Methodology and Methods

### Sample

The study population comprised 300 parents of children attending public elementary schools in grades one through six.

#### Participants’ Characteristics

The majority of teachers, 83.3% (*n* = 250), were female and 16.7% (*n* = 50) were male; In total, 24% (*n* = 72) of the parents were between ages 30 and 39, 69% (207) were between the ages of 40 and 49, 7% (21) were of age 50 or older; 43.3% (130) of the parents held an undergraduate degree, 39.3% (118) held a Master’s degree, 4.7% (14) had a Ph.D. degree, and 12.7% (38) of the participants marked their education as “other.”; 4.3% (13) of the parents were unemployed (homemakers or retirees), 2.3% (7) were seeking employment, 13.3% (40) were business owners or freelancers, and 80% (240) were salaried employees; 2.3% (7) of the parents had one child, 22.7% (68) had two children, 54.7% (164) had three children, 17.7% (53) had four children, and 2.7% (8) of the parents did not indicate the number of children they have; 8.7% (26) of the participants’ children were in first grade, 14% (42) had a child in second grade, 15.3% (46) of the parents had a child in third grade, 23% (69) had a child in fourth grade, 21.3% (64) had a child in fifth grade, and 17.7% (53) had a child in the sixth grade; 10.3% (31) of the participants were living in communal settlements, 14.3% (43) were living on a Moshav, 36% (108) resided in cities, and 39.3 (118) were living on a kibbutz; 28.7% (86) of the participants stated that they lived in the same communal settlement where their child’s teacher lived, 71.3% (214) indicated that they did not live in the same communal settlement as their child’s teacher; 55.7 (167) stated that they and the child’s teacher lived in places governed by the same Regional Council and 44.3% (133) indicated that they did not.

### Instruments

The research instrument used in the current study was an anonymous, self-reporting questionnaire that included three subsections. Responses on the three parts were ranked on a Likert Scale ranging from 1 = “completely disagree” to 5 = “strongly agree.” The subsection about participants’ background variables included nine items: gender, age, education, employment, number of children, grade level of the relevant child, and information about their residential framework.

The first subsection was titled “Attitudes questionnaire regarding parents’ relationship with their child’s school” (Fisher, 2011), and it contains 44 questions that create a scale of parental involvement (α = 0.90). The original questionnaire investigated four variables: improving the school’s resources (α = 0.80), monitoring school processes (α = 0.85), the school’s pedagogy (α = 0.92), and the school’s welfare (α = 0.70). The second subsection of the questionnaire was titled “Questionnaire regarding perceptions of the teacher’s authority” (Adetto, 2012), “which contained 27 statements that create a scale of perceptions of teacher’s authority” (α = 0.75). The third subsection of the questionnaire was titled “questionnaire regarding the level of communality of the settlement/neighborhood.” It was formulated for the current study based on a questionnaire constructed by the Eshchar Company.^1^ This subsection comprises 19 statements that create a scale of communality attributed to a given neighborhood or settlement. The two main variables extracted from the data collected in this subsection were the local authority’s role vis-à-vis its population (α = 0.84) and the participants’ degree of communal involvement (α = 0.82). The list of questionnaire items is shown in Table 1.

**TABLE 1:** The scale of parents’ perception of communality level.

Item no.	Item	Factor I	Factor II
*Factor 1: The local authority’s roles* *(Eigenvalue = 6.48; Explained Variance = 34.13%; α = 0.84; 8 items)*			
88	Residential order and cleanliness	**0.789**	0.080
87	An institution that can address the residents’ problems	**0.704**	0.177
85	The common interests between the residents of the locality / neighborhood	**0.699**	0.151
89	Trustworthy leadership	**0.683**	0.177
82	Personal safety of residents	**0.635**	0.083
84	Residents’ obedience of laws and regulations	**0.632**	0.221
90	Residents’ sense of communality and belonging	**0.538**	0.367
83	Residents’ ability to exert influence in the neighborhood/settlement	**0.478**	0.369
*Factor 2. : Involvement of residents in the community* *(Eigenvalue = 1.78; Explained Variance = 9.35%; α = 0.82; 11 items)*			
73	Engagement in volunteer work for the benefit of the community	0.155	**0.779**
72	Involvement in shared communal activities	0.009	**0.778**
80	Involvement in the neighborhood/settlement’s local council	0.299	**0.641**
77	Holding informal gatherings	0.277	**0.615**
79	Offering each other mutual assistance	0.389	**0.566**
76	Being proud of the neighborhood/settlement	0.167	**0.551**
78	Existence of community social networks	0.139	**0.492**
81	Willingness to pay for shared communal activities	0.359	**0.475**
86	All the residents know each other	0.412	**0.442**
75	Publication of a local newsletter	0.027	**0.441**
74	Taking personal responsibility for the neighborhood/settlement’s cleanliness	0.356	**0.387**

### Data Collection and Analysis

The statistical analyses included the distribution of the responses overall; item analysis and correlation; item-total correlation to exclude items that might be biased or irrelevant to the Scale; and structural equation modeling (SEM), which allows for an examination of complex systems that include numerous variables and relationships among them. Also, the SPSS 21 software and Amos software programs were used to analyze structural equations, which included confirmatory factor analysis (CFA), exploratory factor analysis (EFA), and path analysis. This approach renders models that are more precise than those achieved using traditional variance analysis or multivariable regressions and thus allows for better insight into the causal relationships and the size of the effect of the model’s variables (Ullman and Bentler, 2013).

### The Research Procedure

The research was conducted throughout the 2020–2021 academic year. In the first stage, the research instrument was formulated as a combination of two scales, namely, parental involvement and parental authority, which had been constructed for earlier studies. The communality scale was based on the Eshchar Company’s Construction, which had yet to be used in research. In the second stage, the questionnaires were distributed to a select sample. Initially, the plan was to distribute printed and online questionnaires using social media. Still, with the Corona pandemic outbreak and the college’s closure in March 2020, questionnaires were distributed solely online through social media and parent groups from various towns and settlements. The questionnaires and data gathering were distributed between March and November 2020. Statistical analyses were conducted in December 2020 and January 2021. The research report was written between February and May 2021.

### Adhering to the Rules of Ethics

This study used an anonymous self-reported questionnaire. No identifying data were collected, and the preliminary letter informed potential participants that they were in no way obligated to complete the questionnaire or to provide identifying details. All data were collected solely for the current study and were not shared with any party outside the research team members. The findings are published in a manner that does not disclose participants’ identities.

## Results

### The Parental Involvement Scale

For the parental involvement scale, which included 44 items, Cronbach’s alpha value was 0.90. The Scale rendered four variables, thus matching the structure of the original Scale (Fisher, 2011). Internal reliability testing excluded one item from the original Scale (item 3: “freedom to choose the school for child’s enrollment”). Table 2 presents the distribution of items per variable and the loading for each item.

**TABLE 2:** The scale of parents’ perception of “parental involvement.”

Item no.	Item content	Factor 1	Factor 2	Factor 3	Factor 4
*Factor 1. Monitoring of processes at school* *(Eigenvalue = 8.86; Explained Variance = 20.14%; α = 0.87; 15 items)*					
23	Hiring and firing of school principals	**0.808**	0.052	−0.069	0.005
22	Hiring and firing of school teachers	**0.805**	0.052	−0.047	0.058
24	Assigning teachers to the various classes	**0.729**	0.029	−0.093	0.121
21	Presenting a critique of the curricula to the school management team	**0.653**	0.112	0.258	0.053
26	Criticizing of parents in general	**0.611**	−0.059	0.118	0.253
20	Developing curricula	**0.593**	0.181	0.293	−0.092
19	Representation on pedagogical committees	**0.560**	0.159	0.310	−0.089
36	Sharing in decision making	**0.534**	0.014	0.422	−0.176
10	Expressing an opinion regarding Students’ workload	**0.507**	0.071	0.157	0.169
8	Visiting the classroom during school hours	**0.496**	0.165	0.145	0.108
9	Visiting the school on a weekly basis	**0.480**	0.228	0.073	0.054
35	Holding meetings with the principal regarding school-wide issues	**0.430**	0.217	0.397	−0.247
44	Maintaining weekly contact with the homeroom teacher	**0.403**	0.153	0.122	0.312
33	Awareness of academic achievement levels of their child’s class	**0.394**	−0.192	0.258	0.197
25	Intervening in case of inappropriate teacher behavior	**0.387**	0.108	0.246	0.206
*Factor 2. Supporting school’s resources* *Eigenvalue = 4.86; Explained Variance = 11.03%; α = 0.87; 13 items)*					
14	Responsibility for collecting funds for class activities	0.088	**0.740**	−0.016	0.046
5	Organizing fairs	0.054	**0.729**	−0.062	0.171
2	Participating in the school PTA	0.083	**0.689**	−0.117	0.048
12	Organizing school-wide activities	0.116	**0.676**	0.208	−0.227
6	Assisting in preparing class parties	−0.079	**0.653**	0.040	0.251
1	Participating on the class-level PTA	0.086	**0.642**	−0.216	0.163
16	Responsibility for collecting funds for school-wide activities	0.112	**0.640**	0.020	−0.161
11	Conducting a special lesson in the child’s class	0.043	**0.616**	0.239	−0.160
18	Initiating informal activities	0.152	**0.607**	0.324	−0.133
17	Providing hands-on assistance in the classroom or school	0.065	**0.577**	0.215	0.091
13	Adopting a new immigrant student attending the school	0.071	**0.557**	0.026	−0.043
4	Accompanying the class of field trips	−0.056	**0.519**	−0.030	0.317
15	Funding enrichment programs and special projects	0.211	**0.438**	0.131	0.032
*Factor 3. Awareness of school-related pedagogical processes* *(Eigenvalue = 3.66; Explained Variance = 8.31%; α = 0.85; 7 items)*					
29	Familiarity with the types of social activities that take place in the classroom and in the school	0.014	0.132	**0.756**	0.157
30	Knowledge of the curricula	0.182	0.072	**0.719**	0.193
28	Familiarity with the school’s teaching staff and homeroom teachers	−0.023	0.140	**0.681**	0.314
32	Awareness of violence-related problems	0.135	−0.031	**0.673**	0.308
27	Understanding the social dynamics in the child’s classroom	0.204	0.042	**0.609**	0.275
34	Awareness of decisions made by the teaching staff	0.206	−0.052	**0.596**	0.166
31	Awareness of the population components I the child’s class	0.213	0.060	**0.563**	0.139
*Factor 4. Participation in school-related pedagogical processes* *(Eigenvalue = 3.66; Explained Variance = 5.6%; α = 0.85; 9 items)*					
40	Assistance in preparing for exams	−0.001	0.088	0.204	**0.772**
38	Reviewing notebooks	0.072	−0.011	0.171	**0.729**
37	Assistance with homework preparation	−0.039	0.149	0.221	**0.700**
43	Reviewing exams that have been graded	0.158	0.018	0.205	**0.658**
39	Involvement in addressing discipline-related problems	0.173	−0.172	0.263	**0.594**
42	Involvement when child appeals a grade	0.374	−0.025	0.010	**0.529**
41	Involvement in student-teacher disagreements	0.341	−0.036	0.047	**0.451**
7	Attending parent-teacher meetings	−0.252	0.107	0.144	**0.301**

### The Teacher Authority Scale

For the parents’ perceptions of teacher authority scale, which included 25 items, Cronbach’s alpha value was 0.79. Internal reliability testing led to the exclusion of two items from the original Adetto (2012) scale (item number 57: “teachers should always let students decide for themselves without offering too much guidance”; item 62: “teachers should never think that students must obey rules and norms of behavior only because an authority figure instructed them to do so”). The Scale rendered two main variables, as shown in Table 3.

**TABLE 3:** The scale of parents’ perceptions of the concept of “teacher’s authority.”

Item no.	Item content	Factor I	Factor II
*Factor 1. Teacher’s active listening and empathy* *(Eigenvalue = 4.22; Explained Variance = 24.8%; α = 0.70; 14 items)*			
48	Exercise self-criticism to identify possible mistakes	**0.658**	−0.155
50	Always be consistent in reaction to their Students’ behaviors and actions	**0.591**	
53	Guide their Students’ behaviors and actions and yet be ready to listen and discuss their Students’ ideas.	**0.585**	
64	Always inculcate values and help their students internalize them	**0.569**	0.153
49	Always practice transparency in their actions toward students and the school community	**0.548**	
51	Always be prepared to reconsider a class decision and admit if they made a mistake or accidentally offended a student	**0.546**	
68	Provide their Students’ with clear behavioral guidelines but also understand and accept that there will be disagreements	**0.521**	0.275
46	Set clear guidelines for in-class behavior	**0.504**	0.199
47	Set a personal example in the language spoken and in their day-to-day behavior Act as a role model in terms of their daily language use and behaviors	**0.494**	−0.127
71	Use a soft voice but convey a determined message in their communications with students	**0.443**	0.173
51	Know what is expected of them and be prepared to fulfill these expectations respectfully	**0.428**	0.192
66	Always convey information about the world truthfully and objectively	**0.373**	0.351
70	Must look after their students even if the students are not cooperative	**0.329**	0.164
55	Always consider their Students’ when making decisions, but they need not change their decisions to please the students	**0.250**	0.147
*Factor 2. Behavior and disciplinary issues* *(Eigenvalue = 1.78; Explained Variance = 10.4%; α = 0.72; 11 items)*			
65	Convey a determined message when communicating with students, use a firm tone of voice, and set clear boundaries	0.131	**0.712**
56	Always see to it that students act as instructed without questioning or discussing the instruction	−0.121	**0.695**
54	Always keep a formal distance with students to maintain their authority		**0.579**
61	Consistently enforce their rules and penalize those who do not abide by these rules		**0.573**
67	Always influence and direct Students’ behaviors, actions, and aspirations in the classroom	0.261	**0.512**
59	Always be held responsible for directing and guiding their Students’ behavior	0.283	**0.448**
69	Resolutely enforce the rules they have set for their class	0.341	**0.392**
63	Allow students to make decisions about classroom rules as often as the teachers do.		**0.371**
45	Always be available to address their Students’ questions, requests, and problems	0.271	**0.323**
58	Always supervise and care for their students, not necessarily as equals		**0.300**
60	Always enable students to express their views and perspectives and allow them to make independent decisions	0.158	**0.180**

### The Structural Equation Model

The hypothesis system and the approximate model presented were tested using AMOS software’s structural equation analysis (SEM). Structural equations are the most appropriate analysis method for examining a complex phenomenon and analyzing a system of multivariate relationships, as it is presented graphically in one standard model. This method has advantages over other methods since it allows simultaneous examination of regression equations taking into account measurement errors. An overall evaluation of the model was performed to assess the validity of the theoretical model. The degree of suitability of the general theoretical model for the empirical model was examined (Ullman and Bentler, 2013).

**FIGURE 2:**
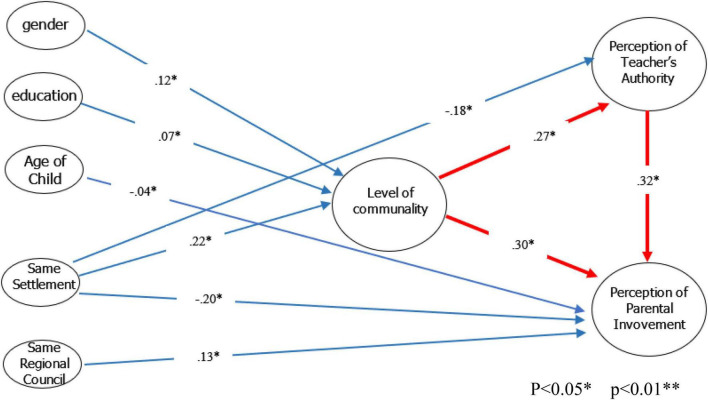
Resulting path coefficients of the proposed research model.

Applying these measures to the current study rendered the following results: RMSEA = 0.007, TLI = 0.995, CFI = 0.99, NFI = 0.904, df = 16, χ^2^ = 16.266, *p* = 0.435. Hence, the value of 1.01 ($\frac{{\text{x}}^{2}}{d\,\text{f}}$) < 3, the TLI and CFI > 0.95, NSI > 0.9, and the RMSEA < 0.1. These measures indicate a good fit between the theoretical and the observed models. Furthermore, these results confirmed the study hypotheses regarding the relationship between the perceived level of communality and the perceived level of parental involvement and the relationship between parents’ perceived level of communality and their perceived level of teacher authority. The resulting path coefficients of the proposed research model are shown in Figure 2.

## Discussion

This study aimed to examine how the level of communality (communal affiliation) affects parents’ perception of children attending public elementary schools, the concept of teacher authority, and the concept of parental involvement.

These issues rendered two main research questions: the first concerned the potential correlation between the degree of communal affiliation of parents residing in different types of residential frameworks and their perceptions of parental involvement, whereas the second question concerned the potential correlation between the degree of communal affiliation of parents residing in different types of residential frameworks and their perceptions of teachers’ authority.

It is important to note that this literature research began with the outbreak of the COVID-19 pandemic. At this time, Israeli society was experiencing uncertainty, and schools had been closed for weeks and even months, such that all study was done remotely. In this emerging reality, it was feared that distance learning and the absence of students from the schools would also distance the parents from the daily school-related activities and thus would affect the study’s outcomes. However, these concerns were allayed once parents’ responses were received and the data were analyzed.

The analysis revealed that parents perceived parental involvement as containing the following four components: monitoring of processes at school, supporting school resources, awareness of pedagogical methods within the school, and active participation in these processes. These findings support the definition of parental involvement as developed by Fisher (2016).

According to the current study’s findings, parents conceptualized teachers’ authority in terms of teachers’ empathy and listening and their behavior toward and discipline required from the students. According to the parents, teachers’ authority manifests in role modeling, setting clear rules and regulations for in-class behavior, and teachers’ admission of their own mistakes. Likewise, demonstrating consistency, understanding, and acceptance of Students’ expressions of disagreement while inculcating moral values was viewed as teachers’ manifestation of authority. These components correspond to those identified in Adetto (2012) definition and recommendations summarized in professional literature reviews regarding the rehabilitation of teachers’ authority (Amit, 2005; Omer, 2018).

The communality scale was composed for the current study and was based on a yet untested questionnaire devised by the Eshchar Company. Accordingly, the concept of communality comprised two factors: the local authority’s functions to benefit the population and the population’s involvement in the community.

Although the three scales were based on scales composed nearly a decade ago, they were nonetheless relevant to the spirit of the times. Moreover, they remained valid and consistent even in a period characterized by emergency conditions and a sense of uncertainty.

The central issue in this research was the ability to predict parental involvement and teachers’ authority by referring to the degree of parents’ sense of communality. As the analysis of the structured equations indicated, there was a good fit between the model proposed initially and the actual findings. All of the study hypotheses were confirmed.

A significant finding that emerged from the structural equation analysis was a strong relationship between parents’ perception of teachers’ authority and their perception of parental involvement. A high score on teachers’ authority predicted a high score on parental involvement (β = 0.32). This finding is surprising, given that studies have shown that what led parents to become involved in their children’s schools was the deterioration in teachers’ status and the erosion of parents’ trust in the teachers (Friedman, 2011). Perhaps, during the Covid-19 pandemic, many parents, particularly with children at the elementary school level, were at home with their children, witnessed the teachers’ performance, and became active partners. Parental involvement was welcomed by both the teachers and the school in general. Having the opportunity to observe the teachers’ work, its complexities personally, and the significant amount of knowledge it requires led parents to appreciate teachers’ degree of professionalism and, as a result, develop positive relationships with them. Consequently, parents who viewed teachers as authoritative figures were more likely to see their involvement as positive (Ice and Hoover-Dempsey, 2011; Adi-Rokach and Greenstein, 2016; Blass, 2020).

Is this a positive change in the relationship between parents and teachers? Perhaps it is related to the ever-growing discussion in Israel and worldwide about the significance of teachers’ and parents’ authority in children’s lives. Parents are beginning to understand that a high degree of teacher authority could benefit their children, contribute to their education, improve their academic achievements, and reduce violence and bullying in the classroom. Hence, parents wish to become involved and help teachers attain these educational goals (Amit, 2009; Dever and Karabenick, 2011; Risanger Sjursø et al., 2019).

The result revealed a relationship between background variables and the level of perceived communality in one’s residential framework (H1). Findings indicated that women perceived communality to be higher than did the men (β = 0.12). Similar conclusions have been found in other studies conducted in Israel and worldwide. Women are more active in the community and fulfill socially significant roles within the community compared to men (Sadan, 2009).

It is interesting to note that this trend has not changed. While it may be interesting to consider why women feel a stronger sense of communality, answering the question is beyond the scope of the current study. However, it may be worth noting that often women are more available than men to spend time with their children in the afternoon hours, which in turn makes them want to feel attached to their community. This topic is important and merits greater attention. It was also found that a higher level of parents’ education predicted a higher level of perceived communality (β = 0.07). It is possible that people with a higher level of education are more frequently recruited to participate in various activities in the community and perhaps even hold key positions in the community, which in turn strengthens their sense of communality (Shadmi-Wortman, 2017). It is also possible that people with a higher level of education feel a stronger sense of self-efficacy and are more able to contribute to and become involved in the community, which is the source of their perceived communality.

The absence of a correlation between the type of residential framework and participants’ sense of communality was surprising (H2). We expected to find that living in smaller residential frameworks, where residents typically engage in various community activities, would correlate with a higher level of perceived communality. Have the kibbutz and the Moshav lost their most conspicuous communal features and became more adjusted to the larger metropolitans’ alienation? It isn’t easy to ascertain whether this is the case; however, it was found that living in the same town or community where their children’s teachers live predicted a high level of perceived communality (β = 0.22). This finding, more than the finding regarding the type of residential framework, reflects the definition of community and communality as presented in the literature review section, according to which a sense of communality can be experienced in any type of settlement. It is the measure of territorial communality that is significant. It enables more interpersonal, face-to-face interactions, which affects the quality of the relationships, and the levels of trust and reciprocity among the residential members. Members of a community have an internal awareness of the collective, distinguishing them from those outside the community (Etzioni, 2000; Theodori, 2005; Lehavi, 2010; Shadmi-Wortman, 2017). It is possible that the traditional sense of communality has been altered over the years, given the changes in Israeli society and perhaps also because of the privatization of the kibbutz framework. Hence, one’s sense of communality no longer depends solely on the type of residential framework where one resides; thus, the concept has expanded to reflect the changes that occurred in Israeli society.

Another hypothesis confirmed by the findings was that parents’ background variables would predict their perception of parental involvement (H4). Furthermore, a negative correlation was found between the age of the children and the parent’s perception of parental involvement (β = 0.04), such that the higher the child’s age, the lower the level of perceived parental involvement. Other studies have found similar finding, which explained that the older the children are, the less they wish to see their parents involved in their school and the more independent they seek to be, and in the same vein, the parents no longer feel that the children need them to be strongly involved in the school (Friedman, 2011; Hornby and Lafaele, 2011). There was no correlation between other background variables and parental involvement or teachers’ authority.

Other interesting findings that emerged and addressed the primary research questions were the strong correlations found between parents’ level of communality and their perceptions of the concepts of teachers’ authority (H7) and parental involvement (H8). Specifically, it was found that a high level of communality predicted a high level of perceived teachers’ authority (β = 0.27) and a high level of parental involvement (β = 0.30) among the parents. According to the professional literature, a high degree of communality indicates a high level of trust and mutuality among community members and other significant institutions and representative community figures. In this context, the teachers and the school represent the latter (Shadmi-Wortman, 2017).

The attempt to explain the correlation between high levels of communality and parents’ high level of involvement is based on the model of “Potential parental involvement,” introduced by Friedman and Fisher (2002). According to this model, identification and involvement with the school are predictors of parental involvement. It may be assumed that parents with a strong sense of communality would also identify strongly with the school. Parents who see the school as playing an essential role in the life of the community are likely to identify with the school’s goals, be more aware of the school’s activities, demonstrate a more positive acceptance of the school’s values and norms, and as a result, have a strong sense of parental involvement.

Another hypothesis examined in this study was that parents’ type of residential framework would predict their perceptions of parental involvement (H6). Although parental involvement did not correlate with parents’ residential kind of framework, residing in the same neighborhood or settlement as their children’s teachers predicted parents’ perceived low levels of parental involvement (β = 0.20). This finding could be explained by the situation that living in the same geographic community offers the conditions for an in-depth familiarity between parents and teachers beyond the formal relationships at school and, consequently, increase parents’ trust in the teachers. The stronger their trust in the teachers is, the less they need to be actively involved in the school (Shechtman and Busharian, 2015).

It is also possible that the sense of togetherness that comes from living in the same neighborhood or settlement makes parents feel that their involvement in the school is redundant. As there is plenty of room for informal interaction between the parents and teachers, for example, in the form of “sidewalk discussions” about the children and the school, the parents see no need for further involvement and hence perceive the level of parental involvement below. The flip side of the same coin is that a prior relationship between teachers and families of students can be a source of teachers’ prejudice about the students, which prevents parents from becoming involved (Caplan, 1995). Relying on a reference from almost two decades ago can corroborate similar cases belonging to the fields of social psychology.

## Data Availability Statement

The original contributions presented in the study are included in the article/supplementary material, further inquiries can be directed to the corresponding author.

## Ethics Statement

Ethical review and approval was not required for the study on human participants in accordance with the local legislation and institutional requirements. Written informed consent from the patients/participants or patients/participants legal guardian/next of kin was not required to participate in this study in accordance with the national legislation and the institutional requirements.

## Author Contributions

Both authors listed have made a substantial, direct, and intellectual contribution to the work, and approved it for publication.

## Conflict of Interest

The authors declare that the research was conducted in the absence of any commercial or financial relationships that could be construed as a potential conflict of interest.

## Publisher’s Note

All claims expressed in this article are solely those of the authors and do not necessarily represent those of their affiliated organizations, or those of the publisher, the editors and the reviewers. Any product that may be evaluated in this article, or claim that may be made by its manufacturer, is not guaranteed or endorsed by the publisher.

## Footnotes

1 A company with expertise in providing social, community, and welfare services. There is no statistical information regarding this scale. Some of the statements were borrowed from it and others were added, to create the current version.
